# Chronic Candida infection, bronchiectasis, immunoglobulin abnormalities, and stunting: a case report of a natural mutation of STAT1 (c.986C>G) in an adolescent male

**DOI:** 10.1186/s12879-020-05734-9

**Published:** 2021-01-07

**Authors:** Yali Yu, Fei Xu, Hui Shen, Jiang Wu

**Affiliations:** 1 Department of Gastroenterology, Zhongnan Hospital, Wuhan University, 169 Donghu Road, Wuchang District, Wuhan City, Hubei Province China; 2 Department of Hematology, Zhongnan Hospital, Wuhan University, 169 Donghu Road, Wuchang District, Wuhan City, Hubei Province China

**Keywords:** Chronic mucocutaneous candidiasis, Bronchiectasis, Immunoglobulin a, Stunting, Gain-of-function mutations, STAT1

## Abstract

**Background:**

Chronic mucocutaneous candidiasis (CMC) is the most common clinical symptom of singer transducer and signal transducer and activator of transcription 1 (STAT1) gain-of-function (GOF) mutations. Bronchiectasis is a chronic lung disease that is characterized by permanent bronchiectasis, causing cough, expectoration, and even haemoptysis. The underlying pathogeny is not yet clear. Immunoglobulin (Ig) A is derived from memory B cells and correlates with immune-related diseases. STAT1 is closely associated with signal transmission and immune regulation.

**Case presentation:**

We report a 17-year-old male patient carrying a GOF mutation in STAT1. The variant led to CMC, bronchiectasis, and elevated serum IgA levels, as well as stunting. Whole-exome sequencing (WES) revealed a c.986C>G (p.P329R) heterozygous mutation in the STAT1 gene.

**Conclusion:**

Further Sanger sequencing analysis of STAT1 in the patient and his parents showed that the patient harboured a de novo mutation.

## Background

The STAT family of proteins are latent cytoplasmic transcription factors. When the proteins are translocated to the nucleus and bind to the corresponding cytokine or growth factor receptor, they are phosphorylated and activated by kinases, interferon, inflammatory factors, epidermal growth factor (EGF) and colony-stimulating factors (CFS) [1–3]. For example, STAT3 is activated by tyrosine phosphorylation in response to EGF and IL-6 [3]; Stat2 becomes phosphorylated after IFN-α treatment [4], and IFN-γ induces tyrosine phosphorylation of STAT91 [5]. The Janus kinase (JAK) family tyrosine phosphorylate STAT family proteins. Activated STAT proteins are translocated to the nucleus to promote transcription, forming the classic JAK-STAT pathway, which is involved in many important biological signal transmissions [6]. There are many members of the STAT family, 7 of which have been clearly defined: STAT1, STAT2, STAT3, STAT4, STAT5a, STAT5b and STAT6 [7, 8]. Among them, STAT1 was the first identified, and it is activated by IFN-α/β [6, 9], which plays an important role in autoimmune diseases [10], such as asthma [11], rheumatoid arthritis [12], and inflammatory bowel disease [13]. Mutations in STAT1 can affect the immune function mediated by IFN-γ and IFN-α/β, thereby affecting host immune defence capacity and increasing susceptibility to mycobacteria, fungi and viruses [14]. STAT1 is both a target of loss-of function (LOF) and GOF mutations [15]. Since STAT1 GOF mutations were first reported in 2011 [16], they have been identified in a growing number of patients and have attracted increasing attention.

GOF mutations in STAT1 are frequently enriched in the coiled-coil.

domain or DNA-binding domain [17], with a variety of clinical manifestations, including CMC infection, autoimmune diseases, and infection leading to early death [18]. Here, we report a case of pathogenic STAT1 GOF mutation in a young male in China with severe, recurrent and persistent pulmonary bacterial infections and aphthous stomatitis since childhood and who then developed bronchiectasis and increased IgA. The patient has undergone many repeated examinations in the past 10 years to confirm the diagnosis. However, genetic analysis was not performed until at age 17, revealing a novel STAT1 GOF mutation (c.986C> G). We propose that patients with unexplained chronic aphthous stomatitis, pulmonary bacterial infections, bronchiectasis and an increase in immunoglobulin IgA may carry STAT1 GOF mutations.

## Case presentation

**Fig. 1:**
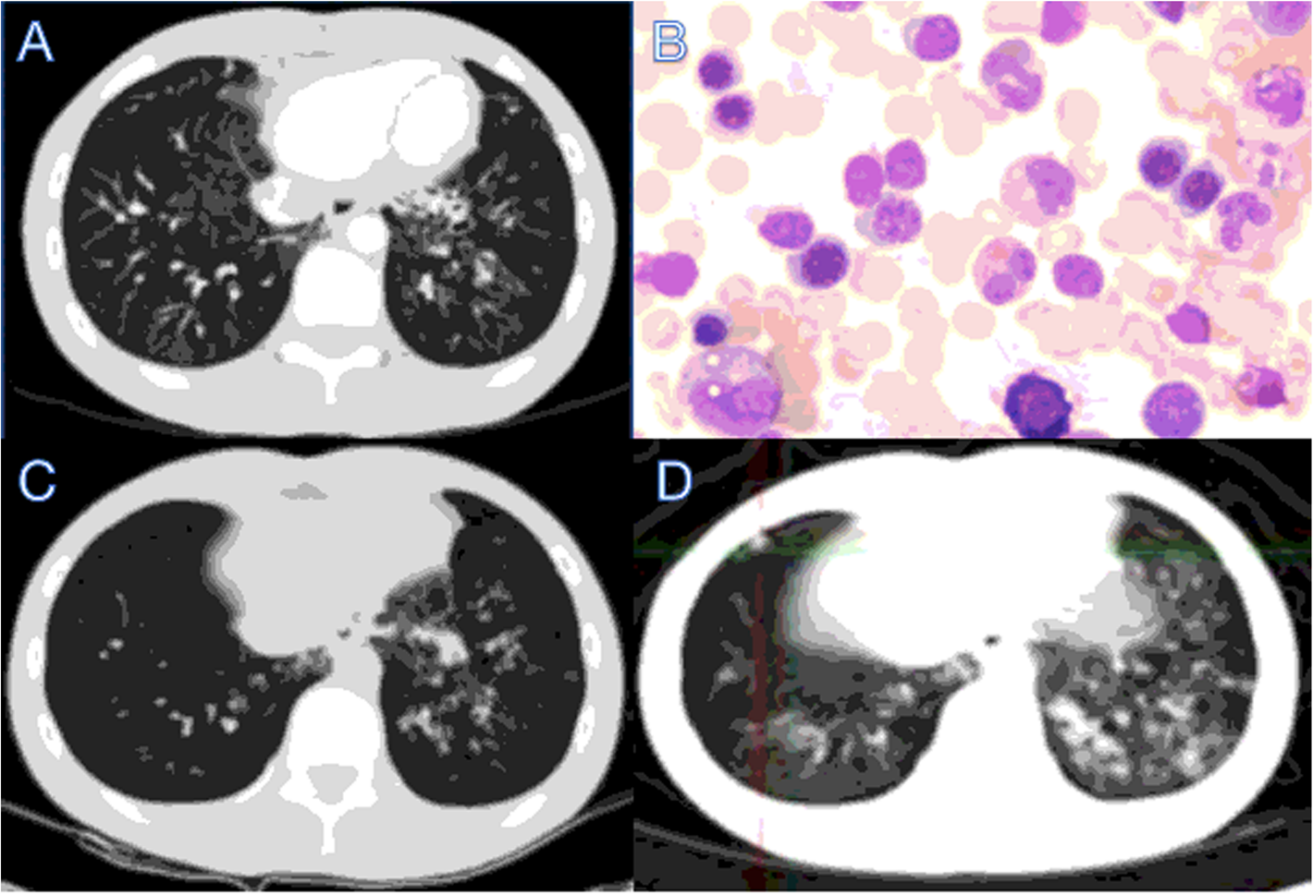
Severe bronchiectasis and bone marrow puncture in the 17-year-old male patient**. a** Bilateral bronchiectasis and bronchiolitis on chest computed tomography scan in the 16-year-old**. b** Cell morphous was observed by microscope of bone marrow puncture**. c** Bilateral bronchiectasis and bronchiolitis on chest computed tomography scan in the 17-year-old**. d** Bilateral bronchiectasis and bronchiolitis on chest PETCT

This case report was approved by Wuhan University Zhongnan Hospital. The 17-year-old patient has been slower than his peers in development and repeatedly experienced pulmonary bacterial infections and aphthous stomatitis since childhood. He became infected with varicella-zoster virus (VZV) and developed lung abscess when he was 10 years old. He was diagnosed with bronchiectasis and elevation of IgA (6.43 g/L) for the first time at the age of 15 years (Fig. 1a). The patient was treated with antiviral and antibiotics treatments discontinuous, and the treatments were interrupted when the symptoms were relieved. At the age of 17, he was 160 cm tall and weighed 40 kg, which was significantly shorter than a male of the same age. He revisited our hospital due to repeated coughing and the symptoms progressed with the treatment of oral antibiotics at home.

Further tests were conducted to determine the underlying pathogenesis, including quantitative serum immunoglobulin immunofixation electrophoresis and autoimmune disease-related antibody assays. We found no abnormality when detecting antibodies related to autoimmune diseases, with no abnormal bands by immunofixation electrophoresis. Therefore, we can rule out the common autoimmune diseases such as rheumatism, systemic lupus erythematosus and Sjogren’s syndrome. Levels of serum IgG (11.70 g/L) and IgM (1.00 g/L) were normal, but those of IgA (6.91 g/L) were elevated. We performed a bone marrow aspiration examination to rule out blood system diseases (Fig. 1b). The patient had repeated coughing; he was diagnosed with bronchiectasis many years ago, but the aetiology was not determined. Therefore, we reperformed chest CT, and the results indicated bronchiectasis (Fig. 1c), which was consistent with his previous diagnosis. At the same time, we performed whole-body positron emission tomography (PET) CT. Despite the presence of bronchiectasis, there were no signs of malignancy (Fig. 1d).

**Fig. 2:**
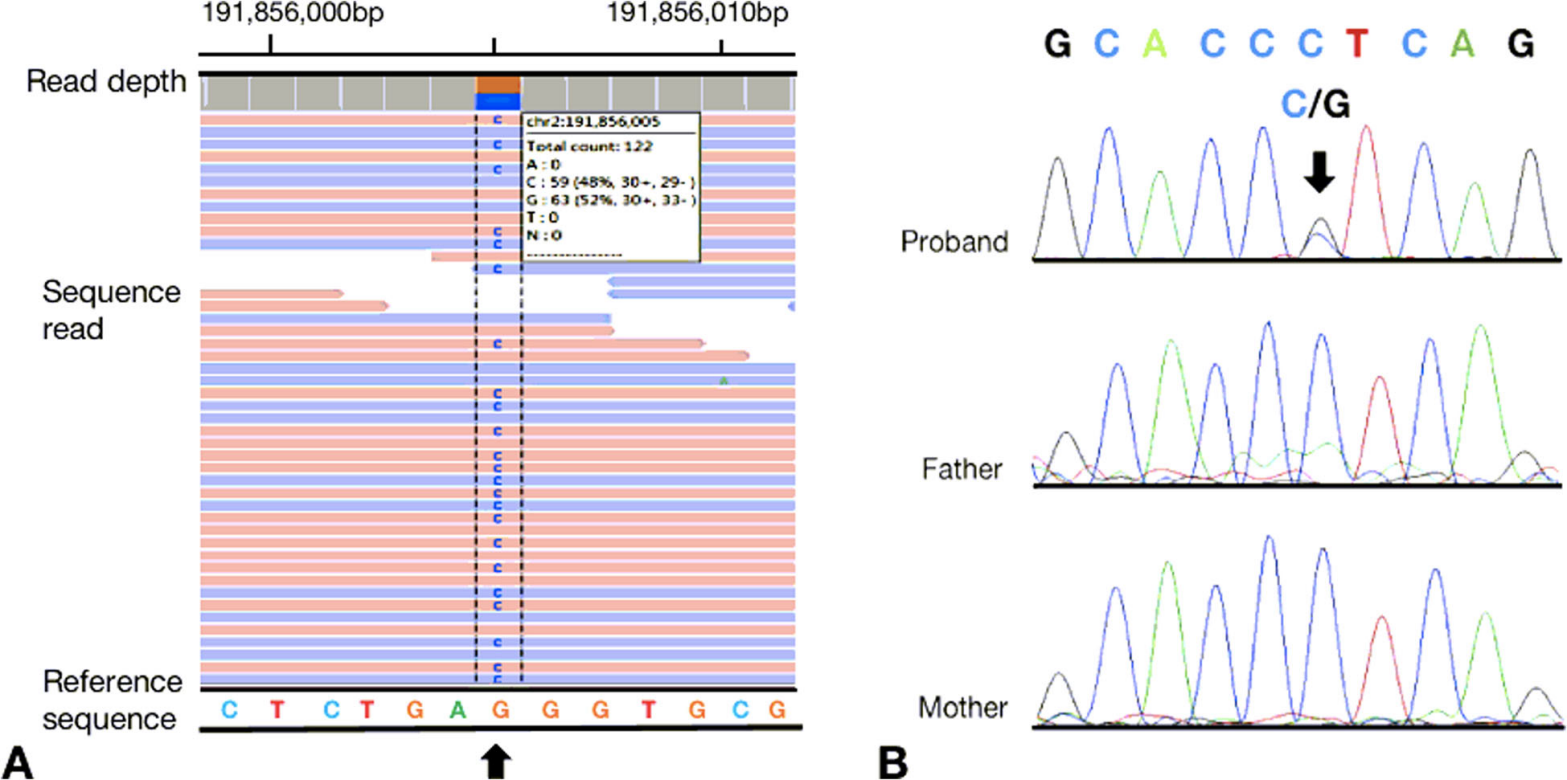
Pathogenic STAT1 variant identified in the 17-year-old male patient. **a** Integrative Genomics Viewer snapshot of the STAT1 pathogenic variant. (NM_007315.3, Exon11, c.986C>G, p.P329R) identified by whole-exome sequencing. **b** Validation of the STAT1 variant by Sanger sequencing. Chromatograms show the heterozygous variant in the proband (patient), indicated by the arrow, and the normal sequence in the unaffected parents

Since the examinations could not clarify the original cause of the patient’s condition and the patient has been ill since childhood, the possibility of genetically related disease was considered, and we further performed WES at the Institute of Hematology, Hematology Hospital, Chinese Academy of Medical Sciences with the consent of the patient’s parents. The report identified a missense variant (NM_007315.3, Exon11, c.986C>G, p.P329R) of the STAT1 gene (chr2:191856005) (Fig. 2a). The inheritance of this mutation type is heterozygous autosomal dominant (AD). The mutation occurs in the STAT DNA-binding domain. According to the American Society of Medical Genetics and Genomics (ACMG) guidelines, the c.986C>G mutation of the STAT1 gene is a suspected pathogenic mutation. The mutation was not found in Human Exon Database (ExAC), Reference Population Thousand Genomes (1000G), or Population Genome Mutation Frequency Database (gnom AD). This mutation occurs at the same position as a mutation that has been determined to be pathogenic, but it involves a different amino acid missense mutation. Based on functional prediction through SIFT (http://sift.jcvi.org/) and Polyphen (http://genetics.bwh.harvard.edu/pph2/), the mutant protein is harmful, leading to immunodeficiency 31C. The disease mainly manifests with repeated respiratory infections, immunodeficiency, immune disorders, recurrent infections, progressive lymphocytopenia, progressive memory B cell reduction, and progressive immunoglobulin reduction. Some patients have hypothyroidism. The disease characteristics are basically consistent with the phenotype of this case, but the level of IgA in this case was elevated. To clarify the origin of the patient’s STAT1 mutation, we performed Sanger sequencing of STAT1 in his parents. As his parents did not carry the variant (NM_007315.3, Exon 11, c.986C>G, p.P329R), this was a de novo mutation (Fig. 2b).

## Discussion and conclusion

With STAT1 GOF mutations, the ability of the protein to respond to IFN-α/β/γ and IL-27 is enhanced, thereby suppressing the differentiation of T cells to IL-17-secreting T cells and decreasing IL-17 secretion. At the same time, STAT1 GOF mutation impairs the pathway by which IL-6, IL-21 and IL-23 induce the differentiation of IL-17 secretory T cells through STAT3, resulting in reduced IL-17 secretion and easily inducing CMC [16, 19]. The most common cause of CMC is a GOF STAT1 variant, with over half of these patients developing CMC [20]. A clinical case study reported in 2016 from France showed that 98% of patients with this type of mutation were infected with CMC [18]. Regarding our case, the patient had repeated aphthous stomatitis since childhood. The heterozygous c.986C>G (p.P329R) variant in STAT1 identified in our patient has not been reported before. This case report describes a new autosomal dominant GOF pathogenic variant in STAT1 that causes CMC.

CMC infection is the most common clinical symptom in patients with STAT1 GOF mutations, and bronchiectasis is common in these patients. In the study of Julie Toubiana’s group, 21% of the patients with STAT1 GOF mutation had bronchiectasis [18]. Impairment of class-switched memory B cells is involved in the pathogenesis of bronchiectasis [21, 22], and studies have reported a reduction in class-switched B cells in patients with STAT1 GOF mutation [23, 24]. Breuer and colleagues proposed that the main cause of bronchiectasis in patients with STAT1 GOF mutant is the reduction of memory B cells, but the links between STAT1 mutation and B cell dysfunction need further study [25]. In general, the level of IgA in patients with STAT1 GOF mutation and bronchiectasis should be decreased, though it was increased in our case. Bronchiectasis may be induced by repeat pulmonary bacterial infections [26], which might have been the pathogenesis of bronchiectasis in our patient.

Patients with STAT1 GOF mutation often experience bacterial infections and invasive fungal infections. Moreover, many patients have autoimmune manifestations, including hypothyroidism, type 1 diabetes, blood cytopenia, and systemic lupus erythaematosus. Invasive infections, cerebral aneurysms, and even cancers can occur. At the same time, many patients have normal results in initial immunologic investigations, such as lymphocyte subpopulations (T, B and NK cells) and immunoglobulin isotypes [18]. Therefore, it is very difficult to determine a patient’s diagnosis based on clinical symptoms or laboratory tests. Accordingly, genetic testing is currently the fastest approach to diagnosing such diseases. The patient in our case had repeated oral fungal and pulmonary bacterial infections since childhood as well as symptoms of immune dysfunction such as bronchiectasis and elevated levels of lgA, consistent with the clinical manifestations of STAT1 GOF mutation. We used WES to identify that the cause of the patient’s disease is STAT1 GOF mutation, and the results clarified that the patient’s variant was not inherited from his parents. This patient has also been stunted since childhood. At 17 years old, he weighed 40 kg, was 160 cm tall, and had a BMI of 15.625 kg/m^2^. The average BMI range of healthy young men is 18.5–23.9 kg/ m^2^. However, the development of his parents is within the range of normal adults, excluding the developmental retardation caused by genetic factors. Then we suspected this STAT1 variant might cause developmental retardation which is worth consideration and further research. This report describes the first patient carrying a pathogenic variant of the STAT1 gene causing CMC infection, bronchiectasis, elevated serum IgA levels and stunting. It is worth noting that WES can serve as an effective detection method to help quickly identify pathogenesis in the presence of diverse unexplainable symptoms.

## Data Availability

The necessary clinical data of the patient are presented in the case report. There is no dataset to be shared in public repositories.

## Abbreviations

CMC: Chronic mucocutaneous candidiasis
STAT1: Singer transducer and activator of transcription 1
GOF: Gain-of-function
Ig A: Immunoglobulin A
WES: Whole exome sequencing
EGF: Epidermal growth factor
CFS: Colony-stimulating factors
AK: Janus Kinase
VZV: Varicella-zoster virus
AD: Autosomal dominant
PET: Positron emission tomography
